# (*E*)-3-(2,3-Dimethoxyphenyl)-1-(2-hydroxy-4-methoxyphenyl)prop-2-en-1-one

**DOI:** 10.1107/S1600536808026949

**Published:** 2008-08-30

**Authors:** Carlos A. Escobar, Andrés Vega, Dieter Sicker, Andrés Ibañez

**Affiliations:** a Universidad Andres Bello, Departamento de Ciencias Químicas, Santiago, Chile; b Institut für Organische Chemie, Universität Leipzig, D-04103 Leipzig, Germany; c Laboratorio de Cristalografía, Departamento de Física, Facultad de Ciencias Físicas y Matemáticas, Universidad de Chile

## Abstract

The mol­ecular conformation of the title compound, C_18_H_18_O_5_, is stabilized by a strong intra­molecular hydrogen bond between the hydroxyl and carbonyl groups. The C=C double bond displays an *E* configuration while the carbonyl group shows an *S-cis* configuration relative to the double bond. The dihedral angle between the two rings is 15.0 (1)°.

## Related literature

For related literature, see: Chu *et al.* (2004 ▶); Desiraju (2002 ▶); Fronczek *et al.* (1987 ▶); Radha Krishna *et al.* (2005 ▶); Rao *et al.* (2004 ▶); Shoja (1999 ▶); Subbiah Pandi *et al.* (2003 ▶); Usman *et al.* (2006 ▶); Wafo *et al.* (2005 ▶); Wallet *et al.* (1995 ▶); Wu *et al.* (2005 ▶).
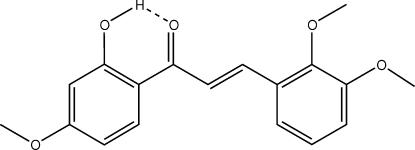


## Experimental

### 

#### Crystal data

C_18_H_18_O_5_
*M* *_r_* = 314.32
Monoclinic, 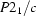

*a* = 4.8793 (5) Å
*b* = 24.283 (3) Å
*c* = 13.0770 (14) Å
β = 97.044 (2)°
*V* = 1537.7 (3) Å^3^
*Z* = 4
Mo *K*α radiation
μ = 0.10 mm^−1^
*T* = 150 (2) K
0.25 × 0.10 × 0.07 mm

#### Data collection

Siemens SMART CCD area-detector diffractometer
Absorption correction: multi-scan (*SADABS*; Bruker, 1999 ▶) *T*_min_ = 0.976, *T*_max_ = 0.993
9482 measured reflections
2717 independent reflections
1522 reflections with *I* > 2σ(*I*)
*R*_int_ = 0.080

#### Refinement

*R*[*F* ^2^ > 2σ(*F* ^2^)] = 0.064
*wR*(*F* ^2^) = 0.139
*S* = 1.03
2717 reflections
212 parameters
H-atom parameters constrained
Δρ_max_ = 0.21 e Å^−3^
Δρ_min_ = −0.16 e Å^−3^

### 

Data collection: *SMART-NT* (Bruker, 2001 ▶); cell refinement: *SAINT-NT* (Bruker, 1999 ▶); data reduction: *SAINT-NT*; program(s) used to solve structure: *SHELXTL-NT* (Sheldrick, 2008 ▶); program(s) used to refine structure: *SHELXTL-NT*; molecular graphics: *SHELXTL-NT*; software used to prepare material for publication: *SHELXTL-NT*.

## Supplementary Material

Crystal structure: contains datablocks I, global. DOI: 10.1107/S1600536808026949/bx2172sup1.cif

Structure factors: contains datablocks I. DOI: 10.1107/S1600536808026949/bx2172Isup2.hkl

Additional supplementary materials:  crystallographic information; 3D view; checkCIF report

## Figures and Tables

**Table 1:** Hydrogen-bond geometry (Å, °)

*D*—H⋯*A*	*D*—H	H⋯*A*	*D*⋯*A*	*D*—H⋯*A*
O20—H20⋯O1	0.84	1.77	2.515 (3)	147

**Fig. 1.**
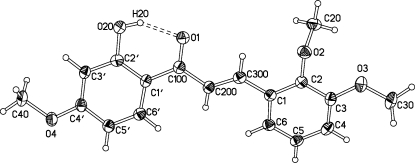
Molecular structure diagramas for I showing numbering scheme. Displacement ellipsoids are at 33% probability level and H atoms are shown as spheres of arbitrary radii.

## supplementary crystallographic information

### Comment

From the synthetic point of view, 2'-hydroxy acetophenones are the choice precursors for the synthesis of 2'-hydroxychalcones trough the Claisen-Schmidt condensation with an aldehyde. Under such basic conditions (*i.e.* KOH), a proton is removed to form the enolate anion at the acetyl moiety. Interestingly, in such a condition the 2'-hydroxyl proton remains unaffected by the base. Deprotonation of this 2'-hydroxy group occurs only under the action of a strong base (*i.e.* hydride) if the methyl ketone's protons in the acetophenone are blocked, as for instance, in the form of a Chalcone.

This behavior is attributed to the intense H-bonding interaction between the 2'-hydroxyl proton and the acetyl moiety of the acetophenone, which is preserved in the derivatives like 2'-hydroxy-chalcones. This structural characteristic of the title compound has been recognized to play a key role in its biological activity and seems to be the basis to its potential as an anti carcinogenic agent. In fact 2'-hydroxychalcones have been found to be cytotoxic against human tumor cells. In the particular case of the title compound this was found to be a potent cytotoxic agent against human lymphocytic and also to monocytic cell linies (Rao *et al.*, 2004). It has been also proved to be a potent antiproliferative agent against tumor cell lines without being more cytotoxic to normal cells (Rao *et al.*, 2004).

The structure of the title compound displays two phenyl rings connected through a three carbon propenone moiety. As shown in Figure 1, one phenyl ring is substituted at positions 2 and 3 with methoxy groups, while the other is substituted at positions 2' and 4' with one hydroxy and one methoxy group respectively.

The hydroxy substitution at 2' produces a six-membered intramolecular O—H···O hydrogen bond with the keto group (Desiraju, 2002). This hydrogen bond is present with almost no exception through the series of compounds with this core, startintg with 2'-Hydroxy-4-methylchalcone (Shoja, 1999). This intramolecular bond leads the carbonyl group to display an S-*cis* configuration in relation to the double bond. The double bond displays an *E* configuration.

The molecule is significantly planar, as reflected in the values determined for the torsion angles. This is also true for molecules substituted with methoxy and/or hydroxy groups at different points of both phenyl sub-systems (Fronczek *et al.*, 1987; Wallet *et al.*, 1995; Subbiah Pandi *et al.*, 2003; Chu *et al.*, 2004; Wafo *et al.*, 2005; Radha Krishna *et al.*, 2005; Wu *et al.*, 2005; Usman *et al.*, 2006).

The packing shows no significant intermolecular hydrogen bonding.

### Experimental

The title compound was prepared as follows: A solution of the 2,3-dimethoxybenzaldehyde, (7.34 mmol in ethanol, 20 ml) was added dropwise to a mixture of 2'-hydroxy-4'-methoxyacetophenone (7.34 mmol, in ethanol, 20 ml) and potassium hydroxide (2 g in 10 ml distilled water) with stirring. The mixture was allowed to react overnight, was then diluted with distilled water (200 ml), neutralized with hydrochloric acid and extracted with ethyl acetate (4 *x* 50 ml). The combined organic phases were concentrated in a rotatory evaporator, redissolved in ethanol and allowed to crystallize, as yellow crystals (31%); mp 98–101 °C.

### Refinement

The hydrogen atoms positions were calculated after each cycle of refinement with *SHELXL* (Bruker,1999) using a riding model for each structure, with C—H distances in the range 0.95 to 0.98 Å. *U*_iso_(H) values were set equal to 1.5*U*_eq_ of the parent carbon atom for methyl groups and 1.2*U*_eq_ for the others. The exception were the hydroxyl hydrogen atom which were located in the Fourier and then refined with the O—H distance constrained to be 0.84 Å and the *U*_eq_ free to refine.

### Crystal data

**Table:**

C_18_H_18_O_5_	*F*_000_ = 664
*M**_r_* = 314.32	*D*_x_ = 1.358 Mg m^−^^3^
Monoclinic, *P*2_1_/*c*	Melting point: 98-101 οC K
Hall symbol: -P 2ybc	Mo *K*α radiation λ = 0.71073 Å
*a* = 4.8793 (5) Å	Cell parameters from 935 reflections
*b* = 24.283 (3) Å	θ = 3.0–19.9º
*c* = 13.0770 (14) Å	µ = 0.10 mm^−^^1^
β = 97.044 (2)º	*T* = 150 (2) K
*V* = 1537.7 (3) Å^3^	Plate, orange
*Z* = 4	0.25 × 0.10 × 0.07 mm

### Data collection

**Table:**

Siemens SMART CCD area-detector diffractometer	2717 independent reflections
Radiation source: fine-focus sealed tube	1522 reflections with *I* > 2σ(*I*)
Monochromator: graphite	*R*_int_ = 0.080
*T* = 150(2) K	θ_max_ = 25.0º
phi and ω scans	θ_min_ = 1.8º
Absorption correction: multi-scan(SADABS; Bruker, 1999)	*h* = −5→5
*T*_min_ = 0.976, *T*_max_ = 0.993	*k* = −28→28
9482 measured reflections	*l* = −15→15

### Refinement

**Table:**

Refinement on *F*^2^	Secondary atom site location: difference Fourier map
Least-squares matrix: full	Hydrogen site location: inferred from neighbouring sites
*R*[*F*^2^ > 2σ(*F*^2^)] = 0.064	H-atom parameters constrained
*wR*(*F*^2^) = 0.139	*w* = 1/[σ^2^(*F*_o_^2^) + (0.0553*P*)^2^] where *P* = (*F*_o_^2^ + 2*F*_c_^2^)/3
*S* = 1.03	(Δ/σ)_max_ < 0.001
2717 reflections	Δρ_max_ = 0.21 e Å^−^^3^
212 parameters	Δρ_min_ = −0.16 e Å^−^^3^
Primary atom site location: structure-invariant direct methods	Extinction correction: none

### Special details

**Table:**

Experimental. 0.3 ° between frames and 30 secs exposure (per frame)
Geometry. All e.s.d.'s (except the e.s.d. in the dihedral angle between two l.s. planes) are estimated using the full covariance matrix. The cell e.s.d.'s are taken into account individually in the estimation of e.s.d.'s in distances, angles and torsion angles; correlations between e.s.d.'s in cell parameters are only used when they are defined by crystal symmetry. An approximate (isotropic) treatment of cell e.s.d.'s is used for estimating e.s.d.'s involving l.s. planes.
Refinement. Refinement of *F*^2^ against ALL reflections. The weighted *R*-factor *wR* and goodness of fit *S* are based on *F*^2^, conventional *R*-factors *R* are based on *F*, with *F* set to zero for negative *F*^2^. The threshold expression of *F*^2^ > σ(*F*^2^) is used only for calculating *R*-factors(gt) *etc*. and is not relevant to the choice of reflections for refinement. *R*-factors based on *F*^2^ are statistically about twice as large as those based on *F*, and *R*- factors based on ALL data will be even larger.

### Fractional atomic coordinates and isotropic or equivalent isotropic displacement parameters (Å^2^)

**Table:**

	*x*	*y*	*z*	*U*_iso_*/*U*_eq_	
C100	0.5970 (6)	0.41587 (14)	0.7398 (2)	0.0353 (8)	
O1	0.6194 (4)	0.45867 (9)	0.68878 (17)	0.0458 (6)	
C200	0.3714 (6)	0.37731 (13)	0.7078 (2)	0.0353 (8)	
H200	0.3422	0.3473	0.7518	0.042*	
C300	0.2072 (6)	0.38252 (12)	0.6203 (2)	0.0375 (8)	
H300	0.2417	0.4132	0.5787	0.045*	
C1	−0.0221 (6)	0.34652 (12)	0.5798 (2)	0.0334 (8)	
C2	−0.1667 (6)	0.35684 (13)	0.4845 (2)	0.0330 (8)	
O2	−0.0892 (4)	0.40002 (9)	0.42551 (16)	0.0438 (6)	
C20	−0.2738 (7)	0.44601 (13)	0.4198 (3)	0.0514 (10)	
H20A	−0.4614	0.4336	0.3951	0.077*	
H20B	−0.2152	0.4735	0.3720	0.077*	
H20C	−0.2713	0.4625	0.4883	0.077*	
C3	−0.3817 (6)	0.32244 (13)	0.4428 (2)	0.0373 (8)	
O3	−0.5036 (5)	0.33541 (9)	0.34667 (17)	0.0504 (7)	
C30	−0.7260 (7)	0.30177 (15)	0.3026 (3)	0.0552 (10)	
H30A	−0.6599	0.2640	0.2951	0.083*	
H30B	−0.7987	0.3163	0.2347	0.083*	
H30C	−0.8726	0.3018	0.3475	0.083*	
C4	−0.4506 (6)	0.27696 (13)	0.4990 (3)	0.0409 (9)	
H4	−0.5951	0.2530	0.4715	0.049*	
C5	−0.3078 (6)	0.26662 (13)	0.5953 (3)	0.0397 (8)	
H5	−0.3561	0.2357	0.6339	0.048*	
C6	−0.0971 (6)	0.30068 (13)	0.6354 (3)	0.0386 (8)	
H6	−0.0009	0.2931	0.7015	0.046*	
C1'	0.7965 (6)	0.40449 (13)	0.8300 (2)	0.0314 (7)	
C6'	0.8088 (7)	0.35443 (13)	0.8848 (2)	0.0381 (8)	
H6'	0.6777	0.3265	0.8635	0.046*	
C5'	1.0022 (6)	0.34452 (13)	0.9674 (2)	0.0387 (8)	
H5'	1.0067	0.3100	1.0018	0.046*	
C4'	1.1930 (6)	0.38540 (14)	1.0010 (2)	0.0377 (8)	
O4	1.3722 (4)	0.37200 (9)	1.08503 (16)	0.0454 (6)	
C40	1.5663 (6)	0.41353 (14)	1.1273 (3)	0.0490 (9)	
H40A	1.6879	0.4235	1.0760	0.074*	
H40B	1.6772	0.3990	1.1890	0.074*	
H40C	1.4652	0.4462	1.1458	0.074*	
C3'	1.1901 (6)	0.43537 (13)	0.9511 (2)	0.0362 (8)	
H3'	1.3179	0.4634	0.9749	0.043*	
C2'	0.9974 (6)	0.44402 (13)	0.8654 (2)	0.0351 (8)	
O20	1.0090 (4)	0.49305 (9)	0.81665 (18)	0.0453 (6)	
H20	0.8938	0.4933	0.7634	0.046 (11)*	

### Atomic displacement parameters (Å^2^)

**Table:**

	*U*^11^	*U*^22^	*U*^33^	*U*^12^	*U*^13^	*U*^23^
C100	0.0270 (18)	0.040 (2)	0.0400 (19)	0.0031 (15)	0.0089 (15)	−0.0042 (16)
O1	0.0407 (14)	0.0398 (15)	0.0549 (15)	−0.0023 (11)	−0.0017 (11)	0.0059 (12)
C200	0.0277 (18)	0.037 (2)	0.0410 (19)	−0.0003 (15)	0.0057 (15)	−0.0033 (16)
C300	0.0309 (19)	0.035 (2)	0.048 (2)	0.0034 (15)	0.0100 (16)	−0.0035 (16)
C1	0.0270 (18)	0.0325 (19)	0.0414 (19)	0.0058 (15)	0.0070 (15)	−0.0060 (15)
C2	0.0237 (18)	0.0351 (19)	0.041 (2)	0.0032 (14)	0.0085 (15)	−0.0037 (16)
O2	0.0394 (14)	0.0424 (14)	0.0498 (14)	−0.0008 (12)	0.0067 (11)	0.0088 (12)
C20	0.054 (2)	0.038 (2)	0.061 (2)	0.0044 (18)	0.0015 (19)	0.0061 (18)
C3	0.0296 (19)	0.045 (2)	0.037 (2)	0.0013 (16)	0.0043 (16)	−0.0064 (16)
O3	0.0408 (14)	0.0594 (17)	0.0487 (15)	−0.0070 (12)	−0.0038 (12)	−0.0063 (13)
C30	0.040 (2)	0.067 (3)	0.057 (2)	−0.009 (2)	−0.0025 (18)	−0.021 (2)
C4	0.033 (2)	0.041 (2)	0.051 (2)	−0.0035 (16)	0.0113 (17)	−0.0148 (17)
C5	0.0344 (19)	0.033 (2)	0.053 (2)	0.0007 (16)	0.0106 (17)	−0.0048 (16)
C6	0.033 (2)	0.0360 (19)	0.047 (2)	0.0051 (16)	0.0060 (16)	0.0003 (17)
C1'	0.0231 (17)	0.039 (2)	0.0342 (18)	−0.0007 (15)	0.0100 (14)	−0.0062 (15)
C6'	0.0313 (19)	0.039 (2)	0.046 (2)	−0.0031 (16)	0.0104 (17)	−0.0017 (16)
C5'	0.0308 (19)	0.043 (2)	0.043 (2)	−0.0002 (16)	0.0077 (16)	0.0013 (17)
C4'	0.0278 (19)	0.046 (2)	0.0392 (19)	0.0019 (16)	0.0048 (16)	−0.0034 (17)
O4	0.0364 (13)	0.0522 (15)	0.0457 (14)	−0.0042 (12)	−0.0020 (11)	0.0029 (12)
C40	0.034 (2)	0.060 (2)	0.051 (2)	−0.0078 (18)	−0.0034 (17)	−0.0113 (19)
C3'	0.0290 (19)	0.040 (2)	0.0400 (19)	−0.0036 (15)	0.0047 (16)	−0.0064 (17)
C2'	0.0337 (19)	0.035 (2)	0.039 (2)	0.0008 (15)	0.0133 (16)	−0.0006 (16)
O20	0.0411 (14)	0.0414 (15)	0.0516 (16)	−0.0057 (11)	−0.0021 (13)	0.0028 (11)

### Geometric parameters (Å, °)

**Table:**

C100—O1	1.247 (4)	O4—C40	1.446 (3)
C100—C1'	1.461 (4)	C3'—C2'	1.388 (4)
C100—C200	1.467 (4)	C2'—O20	1.355 (3)
C200—C300	1.319 (4)	C200—H200	0.9500
C300—C1	1.467 (4)	C300—H300	0.9500
C1—C2	1.377 (4)	C20—H20A	0.9800
C1—C6	1.403 (4)	C20—H20B	0.9800
C2—O2	1.382 (3)	C20—H20C	0.9800
C2—C3	1.398 (4)	C30—H30A	0.9800
O2—C20	1.431 (4)	C30—H30B	0.9800
C3—O3	1.360 (4)	C30—H30C	0.9800
C3—C4	1.391 (4)	C4—H4	0.9500
O3—C30	1.423 (3)	C5—H5	0.9500
C4—C5	1.385 (4)	C6—H6	0.9500
C5—C6	1.372 (4)	C6'—H6'	0.9500
C1'—C6'	1.409 (4)	C5'—H5'	0.9500
C1'—C2'	1.409 (4)	C40—H40A	0.9800
C6'—C5'	1.366 (4)	C40—H40B	0.9800
C5'—C4'	1.394 (4)	C40—H40C	0.9800
C4'—O4	1.357 (3)	C3'—H3'	0.9500
C4'—C3'	1.377 (4)	O20—H20	0.8400
			
O1—C100—C1'	119.7 (3)	C200—C300—H300	116.1
O1—C100—C200	119.4 (3)	C1—C300—H300	116.1
C1'—C100—C200	120.8 (3)	O2—C20—H20A	109.5
C300—C200—C100	122.8 (3)	O2—C20—H20B	109.5
C200—C300—C1	127.7 (3)	H20A—C20—H20B	109.5
C2—C1—C6	118.4 (3)	O2—C20—H20C	109.5
C2—C1—C300	120.1 (3)	H20A—C20—H20C	109.5
C6—C1—C300	121.4 (3)	H20B—C20—H20C	109.5
C1—C2—O2	119.9 (3)	O3—C30—H30A	109.5
C1—C2—C3	121.4 (3)	O3—C30—H30B	109.5
O2—C2—C3	118.7 (3)	H30A—C30—H30B	109.5
C2—O2—C20	114.1 (2)	O3—C30—H30C	109.5
O3—C3—C4	124.5 (3)	H30A—C30—H30C	109.5
O3—C3—C2	116.4 (3)	H30B—C30—H30C	109.5
C4—C3—C2	119.1 (3)	C5—C4—H4	120.1
C3—O3—C30	117.8 (3)	C3—C4—H4	120.1
C5—C4—C3	119.8 (3)	C6—C5—H5	119.7
C6—C5—C4	120.5 (3)	C4—C5—H5	119.7
C5—C6—C1	120.7 (3)	C5—C6—H6	119.6
C6'—C1'—C2'	115.9 (3)	C1—C6—H6	119.6
C6'—C1'—C100	123.8 (3)	C5'—C6'—H6'	118.8
C2'—C1'—C100	120.3 (3)	C1'—C6'—H6'	118.8
C5'—C6'—C1'	122.4 (3)	C6'—C5'—H5'	120.2
C6'—C5'—C4'	119.6 (3)	C4'—C5'—H5'	120.2
O4—C4'—C3'	124.3 (3)	O4—C40—H40A	109.5
O4—C4'—C5'	115.0 (3)	O4—C40—H40B	109.5
C3'—C4'—C5'	120.7 (3)	H40A—C40—H40B	109.5
C4'—O4—C40	118.0 (3)	O4—C40—H40C	109.5
C4'—C3'—C2'	118.9 (3)	H40A—C40—H40C	109.5
O20—C2'—C3'	116.7 (3)	H40B—C40—H40C	109.5
O20—C2'—C1'	120.8 (3)	C4'—C3'—H3'	120.6
C3'—C2'—C1'	122.5 (3)	C2'—C3'—H3'	120.6
C300—C200—H200	118.6	C2'—O20—H20	109.5
C100—C200—H200	118.6		
			
O1—C100—C200—C300	7.8 (5)	C2—C1—C6—C5	0.6 (4)
C1'—C100—C200—C300	−171.6 (3)	C300—C1—C6—C5	−178.4 (3)
C100—C200—C300—C1	179.4 (3)	O1—C100—C1'—C6'	−171.9 (3)
C200—C300—C1—C2	−177.5 (3)	C200—C100—C1'—C6'	7.5 (4)
C200—C300—C1—C6	1.5 (5)	O1—C100—C1'—C2'	6.2 (4)
C6—C1—C2—O2	−176.8 (3)	C200—C100—C1'—C2'	−174.4 (3)
C300—C1—C2—O2	2.3 (4)	C2'—C1'—C6'—C5'	0.2 (4)
C6—C1—C2—C3	−0.7 (4)	C100—C1'—C6'—C5'	178.4 (3)
C300—C1—C2—C3	178.4 (3)	C1'—C6'—C5'—C4'	1.2 (4)
C1—C2—O2—C20	−107.3 (3)	C6'—C5'—C4'—O4	178.5 (3)
C3—C2—O2—C20	76.5 (3)	C6'—C5'—C4'—C3'	−0.7 (5)
C1—C2—C3—O3	−177.7 (3)	C3'—C4'—O4—C40	2.4 (4)
O2—C2—C3—O3	−1.6 (4)	C5'—C4'—O4—C40	−176.8 (3)
C1—C2—C3—C4	0.1 (4)	O4—C4'—C3'—C2'	179.6 (3)
O2—C2—C3—C4	176.2 (3)	C5'—C4'—C3'—C2'	−1.3 (4)
C4—C3—O3—C30	3.2 (4)	C4'—C3'—C2'—O20	−177.7 (3)
C2—C3—O3—C30	−179.0 (3)	C4'—C3'—C2'—C1'	2.8 (4)
O3—C3—C4—C5	178.2 (3)	C6'—C1'—C2'—O20	178.2 (3)
C2—C3—C4—C5	0.5 (4)	C100—C1'—C2'—O20	0.0 (4)
C3—C4—C5—C6	−0.6 (5)	C6'—C1'—C2'—C3'	−2.3 (4)
C4—C5—C6—C1	0.0 (5)	C100—C1'—C2'—C3'	179.5 (3)

### Hydrogen-bond geometry (Å, °)

**Table:**

*D*—H···*A*	*D*—H	H···*A*	*D*···*A*	*D*—H···*A*
O20—H20···O1	0.84	1.77	2.515 (3)	147
